# Altered functional connectivity during face processing in children born with very low birth weight

**DOI:** 10.1093/scan/nsab070

**Published:** 2021-06-18

**Authors:** Julie Sato, Kristina Safar, Marlee M Vandewouw, Nicole Bando, Deborah L O’Connor, Sharon L Unger, Margot J Taylor

**Affiliations:** Diagnostic Imaging, The Hospital for Sick Children, Toronto, ON M5G 1X8, Canada; Psychology, University of Toronto, Toronto, ON M5S 3G3, Canada; Neurosciences and Mental Health, The Hospital for Sick Children, Toronto, ON M5G 0A4, Canada; Diagnostic Imaging, The Hospital for Sick Children, Toronto, ON M5G 1X8, Canada; Neurosciences and Mental Health, The Hospital for Sick Children, Toronto, ON M5G 0A4, Canada; Diagnostic Imaging, The Hospital for Sick Children, Toronto, ON M5G 1X8, Canada; Neurosciences and Mental Health, The Hospital for Sick Children, Toronto, ON M5G 0A4, Canada; Autism Research Centre, Bloorview Research Institute, Holland Bloorview Kids Rehabilitation Hospital, Toronto, ON M4G 1R8, Canada; Institute of Biomedical Engineering, University of Toronto, Toronto, ON M5S 3G9, Canada; Translational Medicine, The Hospital for Sick Children, Toronto, ON M5G 0A4, Canada; Translational Medicine, The Hospital for Sick Children, Toronto, ON M5G 0A4, Canada; Nutritional Sciences, University of Toronto, Toronto, ON M5S 1A8, Canada; Paediatrics, Sinai Health, Toronto, ON M5G 1X5, Canada; Nutritional Sciences, University of Toronto, Toronto, ON M5S 1A8, Canada; Paediatrics, Sinai Health, Toronto, ON M5G 1X5, Canada; Paediatrics, University of Toronto, Toronto, ON M5S 1A8, Canada; Division of Neonatology, The Hospital for Sick Children, Toronto, ON M5G 1X8, Canada; Diagnostic Imaging, The Hospital for Sick Children, Toronto, ON M5G 1X8, Canada; Psychology, University of Toronto, Toronto, ON M5S 3G3, Canada; Neurosciences and Mental Health, The Hospital for Sick Children, Toronto, ON M5G 0A4, Canada; Paediatrics, University of Toronto, Toronto, ON M5S 1A8, Canada; Medical Imaging, University of Toronto, Toronto, ON M5S 1A8, Canada

**Keywords:** preterm, very low birth weight, face processing, MEG, functional connectivity

## Abstract

Structural brain alterations have been reported in key emotional face processing regions following preterm birth; however, few studies have investigated the functional networks underlying these processes in children born with very low birth weight (VLBW). Using magnetoencephalography (MEG), we examined the functional networks related to the implicit processing of happy and angry faces in 5-year-old VLBW (*n* = 28) and full-term (FT; *n* = 24) children. We found that VLBW children showed atypical recruitment of emotional face processing networks in theta (4–7 Hz) compared to FT children. VLBW children showed reduced theta connectivity during processing of angry faces only. This hypo-connected theta-band network was anchored in the left orbitofrontal and parietal regions, involved in the higher level processing of faces and emotion regulation. At the behavioural level, despite VLBW children performing within the normal range, FT children had significantly higher affect recognition scores. Our MEG results suggest a selective impairment in processing angry faces, which would negatively impact social functioning in VLBW children. In FT children, greater recruitment of this theta-band network was positively associated with improved affect recognition scores. Thus, our findings suggest an important role of theta oscillations in early face processing, deficits which may contribute to broader socio-emotional impairments in VLBW children.

## Introduction

A relatively unexplored and crucial facet of social cognitive development in children born with very low birth weight (VLBW, <1500 g) or very preterm (VPT, <32 weeks gestational age [GA]) is the neural networks supporting emotion processing. Behavioural studies have shown that infants born VPT are at an increased risk for long-term socio-emotional impairments. Before school age, VPT children present with difficulties in emotion recognition (Potharst *et al.*, 2013; Witt *et al.*, 2014), which persist into middle childhood, with specific deficits in recognizing angry faces compared to their full-term (FT) peers (Wocadlo and Rieger, 2006; Mossad *et al.*, 2020). Although these difficulties may appear subtle, they have negative downstream effects, including difficulties relating to peers and impaired emotion regulation abilities (Spittle *et al.*, 2009; Boyd *et al.*, 2013; Jones *et al.*, 2013). Further, the inability to recognize facial expressions has been linked with poor social skills in VPT children at school-age (Wocadlo and Rieger, 2006). Despite these reported difficulties, the underlying neural correlates of emotion processing in preterm children are not well understood.

Across development, structural neuroimaging studies have reported volumetric alterations in brain regions involved in emotion processing, including the fusiform gyri (Healy *et al.*, 2013), amygdalae (Peterson *et al.*, 2000), insulae (Nosarti *et al.*, 2014) and frontal regions such as the orbitofrontal cortex (Giménez *et al.*, 2006; Ball *et al.*, 2012). Among the few functional magnetic resonance imaging  (MRI) studies, Scheinost *et al.* (2016) found reduced functional connectivity between the amygdala and other subcortical regions in VPT infants compared to FT controls. These alterations in amygdala functional connectivity persist into adulthood (Papini *et al.*, 2016) and correlate with measures of social competence and socialization (Johns *et al.*, 2019). However, these studies were all conducted at rest (i.e. without the presentation of emotional stimuli) and therefore do not directly probe the networks underlying emotion processing. Further, there remains a critical gap in the literature assessing the functional networks during early childhood, an important transitional period as children enter school, when social difficulties become apparent.

To the best of our knowledge, only one recent study investigated the functional networks underlying emotional face processing in VPT children at 6 and 8 years of age (Mossad *et al.*, 2020). Using magnetoencephalography (MEG), the authors found reduced connectivity in response to angry but not happy faces, in 8-year-old children born VPT compared to controls. These findings suggest relatively spared processing of happy faces, while the neural networks processing negative emotions are altered in school-age children born VPT. This study also highlights the distinct neural networks recruited during the processing of different facial expressions, which cannot be assessed during the resting-state paradigms (Kesler-West *et al.*, 2001; Fusar-Poli *et al.*, 2009; Hoehl *et al.*, 2010). Thus, investigating the early development of these networks should help us understand the heightened risk for a range of behavioural and social problems, as well as an increased prevalence for developing psychiatric disorders (Delobel-Ayoub *et al.*, 2009; Johnson and Marlow, 2011; Treyvaud *et al.*, 2013).

Here, we leveraged MEG to examine whole-brain functional connectivity during the implicit processing of happy and angry faces in VLBW compared to FT children at 5 years of age. MEG is uniquely suited to capture the frequency-specific and rapid interactions within neural networks due to its excellent temporal and spatial resolution. Our secondary aim was to examine relations between connectivity strength in networks of interest and behavioural differences in socio-emotional functioning. Consistent with studies in preterm children (Mossad *et al.*, 2020) and other populations with known socio-emotional impairments (Leung *et al.*, 2014; Safar *et al.*, 2019), we hypothesized that VLBW children would show atypical connectivity in response to angry but not happy faces. Based on the 6-year-old findings from Mossad *et al.* (2020), we predict group differences in the first 300 ms of stimulus onset. Further, we also expect atypical connectivity in VLBW children to include frontal regions known to be important for emotional face processing.

## Methods

### Participants

Participants were a cohort of 52 children born with VLBW [who were recruited as part of a 5-year follow-up (NCT02759809) of a randomized clinical trial (ISRCTN35317141)] and 24 FT controls (recruited via in-hospital advertisements and word of mouth). Exclusion criteria for both groups included a current diagnosis or history of a neurological or neurodevelopmental disorder, chromosomal or major congenital abnormality, uncorrected hearing or visual impairment or colour blindness. For the FT group, exclusion criteria also included a history of preterm birth (<37 weeks GA), brain injury or presence of a learning or language disability. Children were imaged at the Hospital for Sick Children (SickKids), and informed written consent and verbal assent were provided by parents and children, respectively. The study protocol was approved by the SickKids Research Ethics board.

### Emotional faces task and assessments

**Fig. 1.**
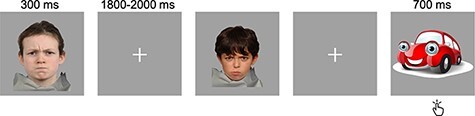
Emotional faces task. Children saw a happy or angry face or a car stimulus (‘catch’ trial). They were instructed to press a button as quickly as possible every time they saw a car and ignore the faces.

Children performed an *implicit* emotional faces task during MEG acquisition (Figure 1), adapted from Batty and Taylor (2006). Children were presented with happy or angry faces for 300 ms, followed by an inter-stimulus interval (fixation cross) that varied between 1800 and 2000 ms. Children were instructed to press a button when a car stimulus (‘catch’ trial, 700 ms duration) appeared on screen and to not respond when face stimuli were presented, thus probing implicit face processing. The task included 130 trials, 50 trials per emotional face (happy and angry) and 30 catch trials. In contrast with the adult faces used by Batty and Taylor (2006), all stimuli included in this study were of school-age children from the NIMH Child Emotional Faces Picture set (Egger *et al.*, 2011) and the Radboud Faces database (Langner *et al.*, 2010) as participants are likely to have more interaction with and receive social-emotional cues from peers of their own age, as well as show more accurate recognition of the faces of own age peer faces compared to other-age peers (Anastasi and Rhodes, 2005).

Outside the MEG scanner, we collected a measure of explicit emotional face processing using the affect recognition subtest of the Developmental Neuropsychological Assessment, Second Edition (Korkman *et al.*, 2007). Parents completed questionnaires to assess the social and emotional functioning of children using the Behaviour Assessment for Children, Third Edition (BASC-3; Reynolds and Kamphaus, 2015) and the Social Responsiveness Scale, Second Edition (SRS-2; Constantino, 2012). The BASC-3 composite scores include externalizing problems, internalizing problems, behavioural symptoms index and adaptive skills. The SRS-2 questionnaire measures the social ability of children and provides a total score. IQ was measured in all children using the Weschler Preschool and Primary Scale of Intelligence IV (Wechsler, 2012). All test scores were standardized to a mean of 100 and standard deviation of 15. Children ‘at risk’ of suboptimal neurodevelopment were classified based on test-specific cut-offs.

### MEG and MRI data acquisition

MEG data were recorded continuously (600 Hz sampling rate and 150 Hz anti-aliasing filter) in a magnetically shielded room using a 151 channel CTF system (CTF MEG International Service Ltd., Coquitlam, BC, Canada) at SickKids hospital. A third-order spatial gradient was used to cancel out external noise. Fiducial coils were placed at left and right pre-auricular points and nasion to record head location continuously. Following the MEG scanning, radio-opaque markers were placed at the same locations as the MEG fiducial coils for co-registration with each participant’s anatomical MRI. T1-weighted MRIs were acquired on a 3T Siemens MAGNETOM PrismaFIT with 20-channel head and neck coil (3D MRPAGE sequence: TR/TE = 1870/3.14 ms, FA = 9°, FOV = 192 × 240 × 256 mm, 0.8 mm isotropic voxels, scan time = 5:03 min).

### MEG pre-processing and source reconstruction

MEG data were pre-processed and analysed using MATLAB software (Mathworks Inc., Natick, MA) and the FieldTrip toolbox (git commit 4c12371; Vinck *et al.*, 2011). Data were epoched into happy, angry and catch trials −1.5 to 3.5 s relative to stimulus onset and filtered offline with a fourth-order two-pass Butterworth band-pass filter from 1 to 150 Hz with a 60 Hz notch filter. Catch trials were not analysed but were reviewed to ensure participants responded during the task (>80% of catch trials). After epoching, independent component analysis was performed in field trip to remove cardiac and ocular artefacts. Epoched trials were also excluded if the MEG sensor signal >2000 fT or if head movement >10 mm from the median head position in any given trial, consistent with previous paediatric MEG studies (Pang, 2011; Taylor *et al.* 2011; Doesburg *et al.* 2013). A single-shell head model was computed based on fiducial positions from each child’s T1-weighted MRI and was normalized onto a standardized ICBM 152 template (Fonov *et al.*, 2011). The cortical and subcortical regions of the automated anatomical labelling (AAL; Tzourio-Mazoyer *et al.*, 2002) atlas were unwarped from the standard MRI brain template space into corresponding locations in each child’s headspace. The linearly constrained minimum variance beamformer (Van Veen *et al.*, 1997) was used to estimate the neural time series at the centroid of each AAL region. The beamformer was run with 5% Tikhonov regularization and correction for centre-of-head bias using the Neural Activity Index.

### Functional connectivity: phase-lag index

The time series data for each AAL region were filtered into theta (4–7 Hz), alpha (8–14 Hz), beta (15–29 Hz) and gamma (30–55 Hz) canonical frequency bands using a two-pass FIR filter. A Hilbert Transform was applied to obtain the instantaneous time series of phase values for each source and frequency band. Functional connectivity between pairs of brain sources was computed using the cross-trial phase-lag index (PLI; Stam *et al.*, 2007), resulting in estimated instantaneous phase synchrony for each of the 90 AAL source-pairs at each sample across the time series. The PLI quantifies the consistent, non-zero phase lag between the time series of two sources and is thus less susceptible to signals associated with volume conduction or beamformer leakage (Stam *et al.*, 2007). PLI values were z-scored at each time point relative to the baseline interval (−200-0 ms relative to stimulus onset) and averaged across the active time window (100–300 ms post-stimulus onset), resulting in a 90 × 90 adjacency matrix. This active window was chosen based on previous findings using a similar task, in which differences in phase synchronization (i.e. connectivity) were reported for emotional face processing during this time window (Leung *et al.*, 2014; Safar *et al.*, 2018, 2019). The average PLI time series, averaged across all subjects, were also visually inspected to confirm task-based changes in connectivity during this time window.

### Statistical analyses

#### Participant characteristics.

Descriptive analyses were conducted for demographic and neuropsychological measures, as well as mean head motion and the number of trials included in the analyses. A *t-*test and Chi-square test were used to determine group differences in continuous and categorical variables, respectively. If the distribution of a continuous variable was non-normal, a non-parametric Mann–Whitney U-test was used instead. Statistical analyses were performed using Statistica (version 7.0; Statsoft Inc., Tulsa, OK, USA). Hypothesis tests were two-tailed and *P *< 0.05 was considered significant.

#### MEG analyses.

The network-based statistic (NBS; Zalesky *et al.*, 2010, 2012) was used to examine between-group MEG differences across and within each emotional face type (happy or angry faces). A 2 × 2 ANOVA with groups (VLBW and FT) as a between-subjects factor and emotion (happy and angry) as a within-subjects factor using NBS was also calculated. Within-group differences for each emotion were also investigated. NBS is a well-established, non-parametric method to identify significant network differences while controlling for the family-wise error rate (FWER) when performing mass univariate testing. In this study, between-group (active windows only, VLBW vs. FT) and within-group (active vs. baseline window) contrasts were computed within each frequency band. The NBS first applies a *t*-test to the normalized PLI values at every connection of the 90 × 90 adjacency matrix, resulting in a *t-*value for each edge. To assign significance at the network level, rather than at the level of individual connections, *t*-values are thresholded and only contiguously connected nodes (i.e. components) that exceed this threshold are subjected to permutation testing. The network components are ascribed a corrected *P*-value by FWER using permutation testing (5000 permutations). To target strong network differences, we thresholded connections at ∼1% of total connections, resulting in a network with 40 edges. Network hubs were identified using node degree, which is the number of connections a node has.

#### Brain–behaviour associations.

Pearson’s correlations were computed between mean connectivity strength in the resulting significant MEG networks with neuropsychological measures and neonatal predictors (birth GA and birthweight).

## Results

### Participant characteristics

Data from 24 VLBW children were excluded from the final analysis due to refusal/inability to complete the MEG task (*n* = 8) or due to insufficient trials (<20 trials per emotion) as a result of excessive artefacts or head motion (*n* = 16). There were no significant differences in demographic or neuropsychological measures between those that were included in the final analysis and those who were not (Supplementary Table S1). Data from all recruited FT children were used. Thus, the final study groups included 28 VLBW children and 24 FT controls. For the VLBW group, 3 out of the 28 (10.7%) children had brain injury at birth (defined as the presence of at least one of the following findings on cranial ultrasound: echodense intraparenchymal lesion, white matter lesion, periventricular leukomalacia, porencephalic cyst or ventriculomegaly with or without intraventricular haemorrhage).

Demographic characteristics and descriptive statistics of neuropsychological measures, head motion and number of trials are summarized in Table 1. Groups did not differ significantly in terms of age at scan (*t*[50] = 1.80, *P *= 0.08) or sex distribution (χ2 [1] = 0.07, *P *= 0.80). Maternal education levels were significantly lower in the VLBW compared to the FT group (χ2 [2] = 11.82, *P *= 0.003). Significant group differences were found for full-scale IQ (*t*[50] = −2.75, *P *= 0.01) and affect recognition (*t*[50] = −2.24, *P *= 0.03), with VLBW children scoring significantly lower than FT controls. However, mean IQ and affect recognition scores for VLBW children were still within the normal range. No group differences were found for BASC-3 or SRS-2 composite scores (*P *> 0.05). There were no significant differences in the proportion of VLBW and FT children who fell within the ‘at-risk’ range for any neuropsychological measures (Supplementary Table S2). A Mann–Whitney U-test was calculated due to non-normality of distributions for mean head motion and number of happy and angry trials. The results revealed no significant group difference in mean head motion (U* *=* *306, *z *=* *−0.54, *P *= 0.59), number of happy (*U *=* *277.5, *z *=* *1.06, *P *= 0.29) or angry trials (*U* = 326.5, *z* = 0.17, *P *= 0.87).

**Table 1.** Demographic characteristics and descriptive statistics

	VLBW group (*n* = 28)	FT group (*n* = 24)	*P*-value
Age at scan (years)	5.8 ± 0.2	5.6 ± 0.4	0.08
Sex (M:F)	15:13	12:12	0.80
Birth weight (grams)	1012 ± 263	3343 ± 521	2.7 × 10^^−26^
Birth GA (weeks)	27.9 ± 2.0	39.5 ± 1.3	2.8 × 10^^−4^
Maternal education level			
High school	8/28 (28.6%)	0/24 (0%)	
University or college	17/28 (60.7%)	14/24 (58.3%)	0.003
Post-graduate training	3/28 (10.7%)	10/24 (41.7%)	
Full-scale IQ	101.6 ± 13.	111.2 ± 11.3	0.01
Affect recognition	103.9 ± 13.6	111.9 ± 11.8	0.03
SRS-2 total score^a^	102.5 ± 13.8	96.1 ± 8.6	0.05
BASC-3 composite scores^b^			
Externalizing problems	102.0 ± 17.1	97.6 ± 9.1	0.26
Internalizing problems	108.2 ± 15.3	101.3 ± 12.5	0.09
Behavioural symptoms index	102.0 ± 16.0	96.2 ± 10.5	0.13
Adaptive skills	105.2 ± 11.4	107.4 ± 9.0	0.45
Mean head motion			
(mm; ± std.)	7.5 ± 2.4	7.7 ± 2.3	0.59
Mean # of trials			
Happy (± std.)	41.0 ± 7.1	40.6 ± 7.7	0.30
Angry (± std.)	38.7 ± 8.1	40.2 ± 8.1	0.87

Categorical variables were presented as frequency (percentage) and continuous variables as mean (standard deviation).

a Higher SRS-2 scores indicate greater social impairments.

b Higher BASC-3 composite scores reflect increased risk of behavioural problems, apart from the adaptive scales, where higher values indicate lower risk.

### Between-group MEG network analyses

**Fig. 2.**
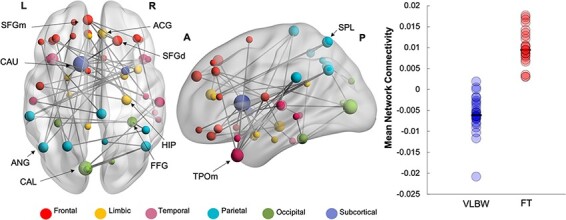
Reduced theta connectivity during face processing in VLBW compared to FT Children (Network 1). Significant group differences, across emotions, were tested in NBS during the active window, 100–300 ms following stimulus presentation (47 edges, 46 nodes, *p_corr_ *= 0.02). Node size is scaled by degree, which is the number of connected edges to the node. The dot plot (right panel) represents the mean network connectivity for VLBW and FT children for this network.

**Fig. 3.**
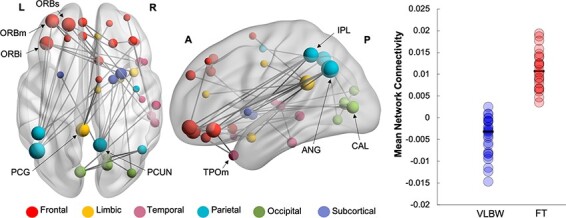
Reduced theta connectivity during angry trials in VLBW compared to FT children (Network 2). Significant group differences were tested in NBS during the active window, 100–300 ms following the presentation of angry faces (40 edges, 37 nodes and *p_corr_ *= 0.005). Node size is scaled by degree. The dot plot (right panel) represents the mean network connectivity for VLBW and FT children for this network.

Group differences at the network level were analysed in NBS within each frequency band. The main effect of group, across emotions, was significant in the theta band only. In this face processing network, hereinafter referred to as ‘Network 1’, VLBW children showed reduced phase synchrony in theta compared to FT controls (47 edges, 46 nodes, *p_corr_* = 0.02; Figure 2). This distributed network included left-lateralized hubs in the middle temporal pole, calcarine and caudate gyri, with other network hubs in frontal and parietal regions (see Supplementary Table S3 for a full list of network nodes). Reduced theta phase synchrony was also found between the left calcarine and the right fusiform gyrus, a key face-processing region. We also ran contrasts within each emotion in the theta band to determine which emotional face type (i.e. happy or angry) was contributing to these group differences. This contrast revealed reduced theta-band phase synchrony during angry face processing in VLBW compared to FT children in ‘Network 2’ (40 edges, 37 nodes, *p_corr_* = 0.005; Figure 3). This hypo-connected network involved frontoparietal connections, anchored in left orbitofrontal (inferior, middle and superior) and parietal hubs (right precuneus, left inferior parietal lobule and angular gyrus) (Supplementary Table S4). There were no significant group differences for happy faces or significant interaction between group and emotion in the theta frequency band.

### Within-group MEG network analysis

**Fig. 4.**
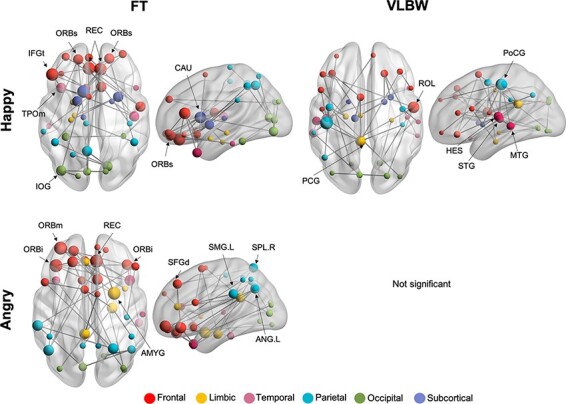
Within-group network analysis during the processing of happy and angry faces in the theta (4–7 Hz) Band. Phase synchrony was examined during the active window (100–300 ms) relative to baseline for each emotional face type. Node size is scaled by degree. FT children showed increased theta connectivity compared to baseline for (a) **Network 3** for happy faces and (b) **Network 4** for angry faces in networks primarily anchored in orbitofrontal regions. (c) **Network 5** for VLBW children showed increased theta connectivity during the processing of happy faces in a dispersed network with hubs in temporal and parietal regions. (d) No significant network was found for angry faces in VLBW children.

Within each group, phase synchrony was examined during the active window for happy and angry faces, separately. In the FT group, increased theta connectivity was found during the processing of both happy faces in ‘Network 3’ (41 edges, 40 nodes, *P < *0.001; Figure 4A) and angry faces in ‘Network 4’ (42 edges, 38 nodes, *P *= 0.002 Figure 4B). Increased theta connectivity to happy faces involved connections in frontal, parietal, subcortical and occipital regions. These connections were primarily anchored in orbitofrontal regions, including the bilateral superior orbital gyri. For angry faces, FT children similarly recruited orbitofrontal regions, such as the left medial and bilateral inferior orbitofrontal nodes. This network involved connections in frontoparietal and limbic regions, including hubs in the left supramarginal gyrus, angular gyrus, superior parietal lobule and right amygdala. In the VLBW group, increased theta connectivity was found during the processing of happy faces only, in ‘Network 5’ (42 edges, 38 nodes, *P = *0.002; Figure 4C). In contrast to the FT networks, VLBW children recruited a more dispersed network with hubs in temporal (left superior and middle temporal gyri), frontal (right Rolandic operculum) and parietal (left postcentral) regions. No significant network was found to angry faces in the VLBW group.

### Brain–behaviour correlation

Mean connectivity strength was extracted for each significant network (1–5) and correlated with neuropsychological measures and neonatal predictors. These associations were conducted separately for each group. In the FT group, mean connectivity strength in Network 2 was positively correlated with affect recognition scores (*r = *0.424, *p*_uncorr_ = 0.039; Supplementary Figure S1). This correlation was not significant in the VLBW group (*r = *0.128, *p*_uncorr_ = 0.516). No other significant brain**–**behaviour correlations were found in either group.

## Discussion

In the present study, we demonstrate that children born with VLBW show atypical recruitment of emotional face processing networks compared to FT controls. These between-group differences were confined to the theta frequency band and driven by the angry faces. Reduced theta connectivity during angry face processing was found in a network anchored in left orbitofrontal and parietal regions in VLBW compared to FT children. Furthermore, mean connectivity strength in this network (Network 2) was significantly correlated with affect recognition scores in the FT group only, suggesting that that greater recruitment of this network is associated with improved explicit face processing. We also analysed the functional networks underlying happy and angry face processing within each group. These results revealed significant networks in theta during the processing of happy and angry faces in FT children, while VLBW children showed increased theta connectivity to happy faces only. At the behavioural level (i.e. outside the scanner), despite mean IQ and affect recognition scores falling within the average range for children born with VLBW, the FT group had significantly higher scores for both these measures. We found no significant group difference in parent-reported measures of social skills or behaviour.

Our between-group findings revealed that across emotions, children born with VLBW show reduced theta connectivity during face processing (Network 1). This distributed network included major hubs in temporal and early visual areas, as well as in frontal and parietal areas. This hypo-connected network also involved the bilateral fusiform gyri, a brain region specialized in face perception (McCarthy *et al.*, 1997). This finding of hypo-connectivity during face processing involving the fusiform gyri is consistent with a recent MEG study in VPT children (Mossad *et al.*, 2020). The authors found that 8-year-old VPT compared to FT children showed reduced connectivity in response to angry faces within the theta and gamma frequency bands. Further, structural alterations of the fusiform gyri have been reported in adolescents born VPT (Nosarti *et al.*, 2008) and correlate with socialization difficulties in young adulthood (Healy *et al.*, 2013). Together, these findings suggest a vulnerability of the fusiform gyri, both structurally and functionally, that is present at preschool age and persists into young adulthood. Altered theta connectivity involving the fusiform gyrus may thus contribute to impairments in face processing observed in this population, which is supported by the lower affect recognition scores in our sample of VLBW children compared to FT controls.

To further investigate which emotion was driving these network differences, we examined group differences within happy and angry faces separately. We found that VLBW compared to FT children showed reduced theta connectivity in response to angry faces only (Network 2). This hypo-connected network to angry faces involved frontoparietal connections anchored in the left orbitofrontal cortex. The network also included the left posterior cingulate cortex and right precuneus, regions implicated in emotional evaluation (Wright *et al.*, 2008). In contrast to the early face-recognition systems in temporal and occipital areas, the orbitofrontal cortex is involved in the higher level processing and regulation of emotions (Haxby *et al.*, 2002; Rolls, 2004). Emotion regulation deficits and structural alterations of the orbitofrontal cortex have been reported in preterm samples at infancy, childhood and adolescence (Giménez *et al.*, 2006; Clark *et al.*, 2008; Ball *et al.*, 2012; Urbain *et al.*, 2019). The ability to appropriately suppress unwanted behaviours, especially in response to negative emotions, is critical to successful peer interactions. This is supported by behavioural studies in FT children linking poorer social competence with lower accuracy at recognizing facial expressions, particularly angry faces (Maxim and Nowicki, 2003). Given the high risk for social difficulties, as well as an increased incidence of psychiatric morbidities beginning in early childhood in children born with VLBW, our findings indicate a potential underlying contributor. We also found that higher connectivity during angry face processing was associated with improved affect recognition scores in FT but not VLBW children. These results provide further support that recruitment of this network is important for both implicit and explicit emotional face processing, specifically for angry faces.

There were no significant group differences in any other frequency band, suggesting that theta oscillations may underlie face processing difficulties commonly reported in this population. Theta, a slow-wave frequency band, is involved in long-range communication between brain regions (Von Stein *et al.*, 2000). Although most frequently implicated in learning and memory, the theta band has also been associated with affective functions, such as face recognition (Güntekin and Başar, 2014). Importantly, our results are consistent with previous work in preterm-born children showing altered recruitment of slow-wave frequency bands during emotional face processing (Mossad *et al.*, 2020). In addition, resting-state MEG studies have also reported altered theta-band connectivity in VPT children (Ye *et al.*, 2015), which was correlated with externalizing behaviour and behavioural regulation (Kozhemiako *et al.*, 2019). Thus, together our results suggest an important role of theta oscillations in early face processing, which may contribute to broader impairments in socio-cognitive functioning in children born with VLBW.

Our within-group results showed that FT children engaged similar networks during the processing of happy (Network 3) and angry (Network 4) faces. However, one notable difference was the recruitment of the right amygdala during angry face processing, consistent with right-hemisphere dominance when processing negative emotions (Lanteaume *et al.*, 2007). The amygdalae play a key role in emotion processing, including directing attention to salient emotional stimuli (Adolphs, 2002). In contrast to the FT group, VLBW children only showed increased theta connectivity to happy faces (Network 5). This finding is in line with the protracted maturational course of emotional face recognition, with happy faces being recognized earlier compared to angry faces (Markham and Adams, 1992; Gao and Maurer, 2010). While these results suggest a maturational delay in the networks recruited for angry face processing, previous studies have shown that these impairments persist into adulthood with individuals born VPT showing reduced accuracy at recognizing anger at low-intensity levels (Papini *et al.*, 2016). Another recent study found that these differences in processing angry faces continue into adulthood in those born extremely LBW and were linked to frontal EEG asymmetry (Amani *et al.*, 2020). Thus, further studies are necessary to understand the maturation of these emotional face processing networks, as well as developmental periods that may be amenable to intervention.

In summary, we investigated functional connectivity during emotional face processing using MEG and found decreased theta connectivity to angry faces in children born with VLBW. This hypo-connected network was anchored in the left orbitofrontal cortex, a key emotion processing region with links to inhibitory control. The ability to process negative emotions and respond appropriately is critical to successful social interactions and may be particularly impaired in children born with VLBW, despite normal scores on other standardized measures. Importantly, higher mean connectivity strength in this angry face processing network was associated with improved affect recognition in FT but not VLBW children. Further, despite our behavioural findings suggesting overall poorer affect recognition in VLBW compared with FT children, our MEG findings demonstrated atypical recruitment of regions only underlying angry face processing. Future studies should complete longitudinal investigations to determine the maturation of these emotional face processing networks and how they contribute to impairments in the social functioning of children born with VLBW.

## Supplementary Material

nsab070_Supp
