# Supplementary material for: Altered functional connectivity during face processing in children born with very low birth weight
Source: Soc Cogn Affect Neurosci. 2021 Jun 18;16(11):1182–90. doi: 10.1093/scan/nsab070 (PMC8599272; doi:10.1093/scan/nsab070)
Supplement: nsab070_Supp [file nsab070_supp.zip › scan-21-040-File007.docx]

**Altered functional connectivity during face processing in children born very low birth weight.**

**Supplementary Materials**

**
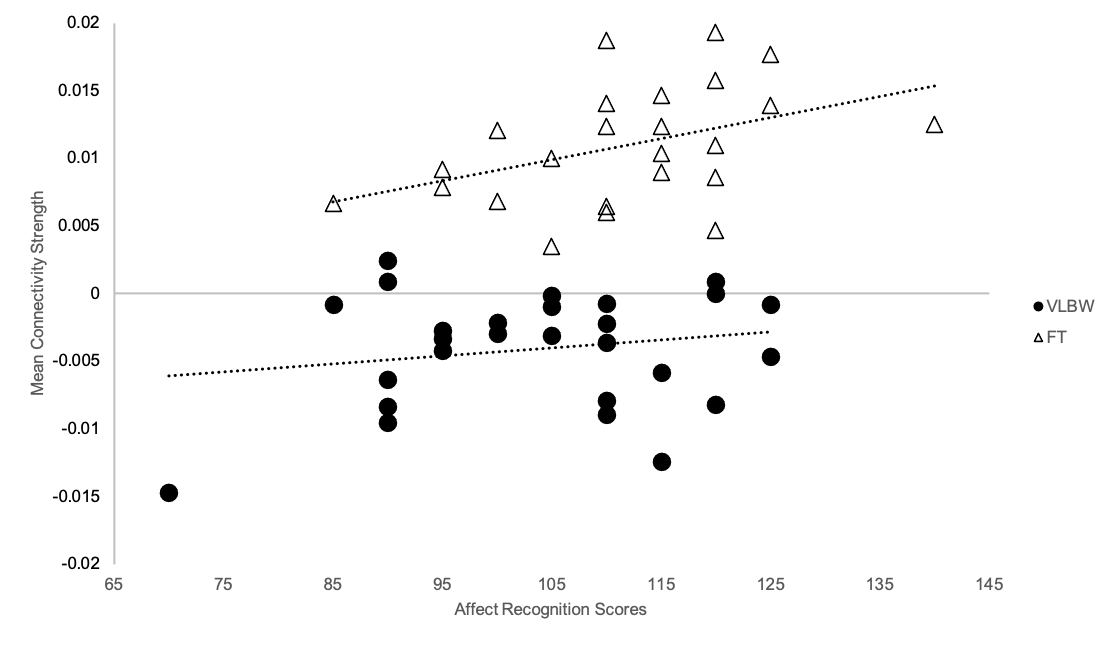
**

**Supplemental Figure S1. Correlation between mean theta network connectivity during angry face processing and affect recognition scores in VLBW and FT children.** A significant positive correlation was found in the FT group (white triangles) between higher mean connectivity strength in **Network 2** in theta and improved affect recognition scores (*r=*0.424, *p_uncorr_*=0.039). This correlation was not significant in the VLBW group (black dots; *r=*0.128, *p_uncorr_*=0.516).

**Table S1.** Comparison of demographic and neuropsychological measures of VLBW children included in the final analyses and those who were not.

|  | **VLBW children included in final analyses (n=28)** | **VLBW children not included in final analyses (n=24)** | ***p*-value** |
| --- | --- | --- | --- |
| Age at scan (years) | 5.8 ± 0.2 | 5.7 ± 0.2 | 0.02 |
| Sex (M:F) | 15:13 | 13:11 | 0.97 |
| Birth weight (grams) | 1012 ± 263 | 1009 ± 243 | 0.96 |
| Birth gestational age (weeks) | 27.9 ± 2.0 | 28.1 ± 2.6 | 0.80 |
| Maternal education level  High School  University or College  Post-graduate training | 8/28 (28.6%)  17/28 (60.7%)  3/28 (10.7%) | 5/22 (22.7%)*  14/22 (63.6%)  3/22 (13.6%) | 0.94 |
| Full-scale IQ | 101.6 **±** 13.5 | 96.8 ± 17.8 | 0.27 |
| Affect Recognition | 103.9 **±** 13.6 | 96.6 ± 24.2 | 0.18 |
| SRS-2 total score | 102.5 **±** 13.8 | 108.0 ± 13.8 | 0.16 |
| BASC-3 composite scores  Externalizing problems  Internalizing problems  Behavioural symptoms index  Adaptive skills ^a^ | 102.0 **±** 17.1  108.2 **±** 15.3  102.0 **±** 16.0  105.2 **±** 11.4 | 102.8 ± 17.1  109.6 ± 21.8  102.8 ± 15.0  101.8 ± 13.9 | 0.86  0.78  0.86  0.33 |

Comparisons between study participants and those who did not participate were analyzed by using independent samples t tests for continuous data and chi-square tests for categorical data. *Missing maternal education level for two participants.

**Table S2.** Comparison of ‘at-risk’ neurodevelopmental scores of VLBW and FT children.

| **“At risk” scores** | **VLBW group (N=28)** | **FT group (N=24)** | ***p*-value** |
| --- | --- | --- | --- |
| Full-scale IQ, <80  Affect recognition, <80  SRS-2 total score, ≥115  Externalizing problems, ≥115  Internalizing problems, ≥115  Behavioural symptoms index, ≥115  Adaptive skills, ≤85 | 1/28 (3.6%)  1/28 (3.6%)  5/28 (17.9%)  4/28 (14.3%)  8/28 (28.6%)  5/28 (17.9%)  2/28 (7.1%) | 0/24 (0%)  0/24 (0%)  2/24 (8.3%)  2/24 (8.3%)  4/24 (16.7%)  1/24 (4.2%)  0/24 (0%) | 1.0  1.0  0.44  0.67  0.35  0.20  0.49 |

Comparisons in the proportion of VLBW and FT children that fell within the ‘at-risk’ range were analyzed by using chi-square tests.

**Table S3.** Brain regions and associated number of connections involved in “Network 1” during face processing in children born VLBW (47 edges, 46 nodes).

| **Brain regions (nodes)** | **Number of connections (degree)** |
| --- | --- |
| \| Frontal_Sup_L \| \| --- \| \| Frontal_Sup_R \| \| Frontal_Sup_Orb_L \| \| Frontal_Mid_L \| \| Frontal_Mid_R \| \| Frontal_Inf_Tri_L \| \| Frontal_Inf_Orb_L \| \| Frontal_Inf_Orb_R \| \| Rolandic_Oper_L \| \| Supp_Motor_Area_L \| \| Olfactory_R \| \| Frontal_Sup_Medial_L \| \| Rectus_L \| \| Rectus_R \| \| Insula_R \| \| Cingulum_Ant_L \| \| Cingulum_Ant_R \| \| Cingulum_Post_L \| \| Hippocampus_R \| \| ParaHippocampal_L \| \| Amygdala_L \| \| Amygdala_R \| \| Calcarine_L \| \| Calcarine_R \| \| Cuneus_L \| \| Fusiform_L \| \| Fusiform_R \| \| Parietal_Sup_L \| \| Parietal_Inf_R \| \| SupraMarginal_L \| \| Angular_L \| \| Angular_R \| \| Precuneus_R \| \| Paracentral_Lobule_R \| \| Caudate_L \| \| Putamen_R \| \| Heschl_R \| \| Temporal_Sup_L \| \| Temporal_Sup_R \| \| Temporal_Pole_Sup_L \| \| Temporal_Pole_Sup_R \| \| Temporal_Mid_L \| \| Temporal_Mid_R \| \| Temporal_Pole_Mid_L \| \| Temporal_Pole_Mid_R \| \| Temporal_Inf_R \| | \| 1 \| \| --- \| \| 3 \| \| 1 \| \| 2 \| \| 2 \| \| 1 \| \| 2 \| \| 1 \| \| 2 \| \| 1 \| \| 1 \| \| 3 \| \| 2 \| \| 2 \| \| 2 \| \| 1 \| \| 3 \| \| 1 \| \| 3 \| \| 1 \| \| 1 \| \| 2 \| \| 5 \| \| 1 \| \| 1 \| \| 2 \| \| 3 \| \| 3 \| \| 2 \| \| 3 \| \| 3 \| \| 3 \| \| 1 \| \| 3 \| \| 6 \| \| 2 \| \| 1 \| \| 2 \| \| 3 \| \| 1 \| \| 1 \| \| 1 \| \| 1 \| \| 4 \| \| 3 \| \| 1 \| |

**Table S4.** Brain regions and associated number of connections involved in “Network 2” during angry face processing in children born VLBW (40 edges, 37 nodes).

| **Brain regions (nodes)** | **Number of connections (degree)** |
| --- | --- |
| \| Precentral_R \| \| --- \| \| Frontal_Sup_L \| \| Frontal_Sup_R \| \| Frontal_Sup_Orb_L \| \| Frontal_Sup_Orb_R \| \| Frontal_Mid_R \| \| Frontal_Mid_Orb_L \| \| Frontal_Mid_Orb_R \| \| Frontal_Inf_Orb_L \| \| Frontal_Inf_Orb_R \| \| Supp_Motor_Area_R \| \| Olfactory_R \| \| Frontal_Sup_Medial_L \| \| Rectus_L \| \| Rectus_R  Insula_R  Cingulum_Ant_R \| \| Cingulum_Mid_R \| \| Cingulum_Post_L  Amygdala_R  Calcarine_L \| \| Calcarine_R \| \| Lingual_R \| \| Occipital_Mid_R \| \| Parietal_Inf_L \| \| Angular_L \| \| Angular_R \| \| Precuneus_R \| \| Putamen_L \| \| Putamen_R \| \| Pallidum_R \| \| Heschl_R \| \| Temporal_Sup_R \| \| Temporal_Pole_Sup_R \| \| Temporal_Mid_R \| \| Temporal_Pole_Mid_R \| \| Temporal_Inf_R \| | \| \| 1 \| \| --- \| \| 1 \| \| 2 \| \| 4 \| \| 1 \| \| 2 \| \| 4 \| \| 1 \| \| 4 \| \| 1 \| \| 1 \| \| 2 \| \| 2 \| \| 2 \| \| 1 \| \| 2 \| \| 1 \| \| 1 \| \| 4 \| \| 2 \| \| 3 \| \| 3 \| \| 2 \| \| 2 \| \| 4 \| \| 5 \| \| 2 \| \| 4 \| \| 1 \| \| 3 \| \| 2 \| \| 1 \| \| 2 \| \| 1 \| \| 2 \| \| 2 \| \|  \| \| \| --- \| --- \| --- \| --- \| --- \| --- \| --- \| --- \| --- \| --- \| --- \| --- \| --- \| --- \| --- \| --- \| --- \| --- \| --- \| --- \| --- \| --- \| --- \| --- \| --- \| --- \| --- \| --- \| --- \| --- \| --- \| --- \| --- \| --- \| --- \| --- \| --- \| --- \| |
